# Supplementary material for: Identification of photoperiod-induced specific miRNAs in the adrenal glands of Sunite sheep (Ovis aries)
Source: Front Vet Sci. 2022 Jul 22;9:888207. doi: 10.3389/fvets.2022.888207 (PMC9354845; doi:10.3389/fvets.2022.888207)
Supplement: Supplementary file 1 [file Data_Sheet_1.ZIP › Supplementary file/miRDeep2 file.zip!/miRDeep2 file/Novel_Structure/LP42A2/Novel_6.pdf]

Provisional ID	:	NC_019470.2_22952
Score total	:	99.1
Score for star read(s)	:	3.9
Score for read counts	:	91.4
Score for mfe	:	2.2
Score for randfold	:	1.6
Score for cons. seed	:	
Total read count	:	191
Mature read count	:	183
Loop read count	:	0
Star read count	:	8

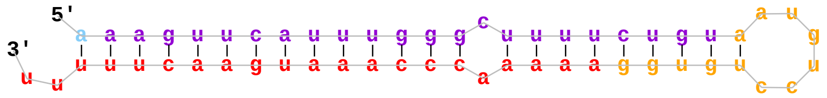

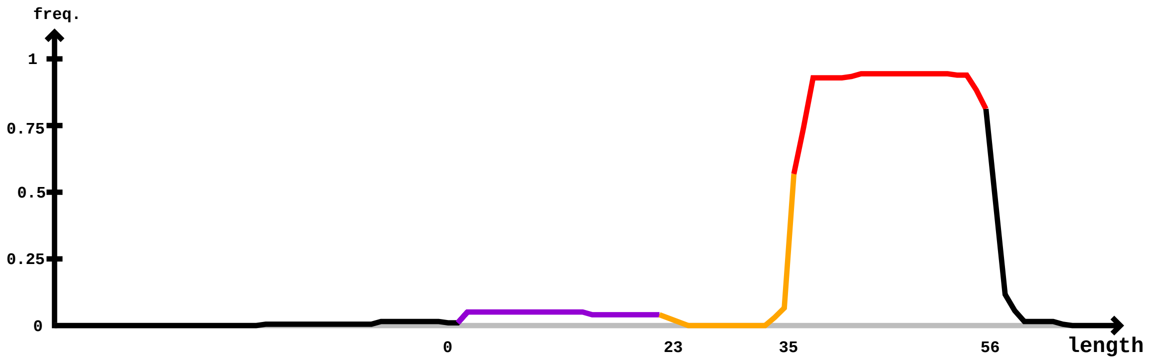

Star Mature

```
5'- acuuagcagcagcaguaguccauuguauuugguuggccaaaaaguucauuugggcuuuucuguaauguccugugg aaaaacccaaaugaacuuuuuggccaaccaagca  -3'  obs
    acuuagcagcagcaguaguccauuguauuugguuggccaaaaaguucauuugggcuuuucuguaauguccuggaaaaacccaaaugaacuuuuuggccaaccaagca         exp
    ...........(((((.....))))).(((((((((((((((((((((((((((.(((((((((......))))))))).)))))))))))))))))))))))))))..  reads  mm  sample
    ....................cauuguauuugguuggcca......................................................................  1      0   seq
    ................................uuggGcaaaaaguucauuuggg.......................................................  2      1   seq
    .........................................aaguucauuugggcuuuucugu..............................................  7      0   seq
    .........................................aaguucauuugggcuuuucuAu..............................................  1      1   seq
    .........................................................................Ugaaaaacccaaaugaacuuu...............  2      1   seq
    .........................................................................ggaGaaacccaaaugaacuuu...............  1      1   seq
    .........................................................................ggaaaaacccGaaugaacuuu...............  1      1   seq
    .........................................................................Ugaaaaacccaaaugaacuuuu..............  1      1   seq
    .........................................................................ggaaaaacccaaaugaacuuuA..............  1      1   seq
    ..........................................................................gaaaaacccaGaugaacuuuu..............  6      1   seq
    ..........................................................................gaaaaacccaGaugaacuuuuu.............  1      1   seq
    ...........................................................................aaaaacccaGaugaacu.................  1      1   seq
    ...........................................................................aaaaacccaGaugaacuuu...............  7      1   seq
    ...........................................................................aaaaacccGaaugaacuuuu..............  2      1   seq
    ...........................................................................aaaaacccaGaugaacuuuu..............  4      1   seq
    ...........................................................................aaaaacccaaaugaacuuuuG.............  1      1   seq
    ...........................................................................aaaaGcccaaaugaacuuuuu.............  2      1   seq
    ...........................................................................aaaaacccaaaugaacuuuuA.............  2      1   seq
    ...........................................................................aaaaacccaGaugaacuuuuu.............  42     1   seq
    ...........................................................................Caaaacccaaaugaacuuuuu.............  1      1   seq
    ...........................................................................aaaaCcccaaaugaacuuuuu.............  2      1   seq
    ...........................................................................aaaaacUcaaaugaacuuuuu.............  1      1   seq
    ...........................................................................aaaaacccaaaugaacuuuuuU............  2      1   seq
    ...........................................................................Caaaacccaaaugaacuuuuug............  3      1   seq
    ...........................................................................aaaaacccaGaugaacuuuuug............  2      1   seq
    ...........................................................................aaaaacccaaaugaacuuuuuA............  6      1   seq
    ...........................................................................aaaaGcccaaaugaacuuuuug............  6      1   seq
    ...........................................................................aaaaacUcaaaugaacuuuuug............  9      1   seq
    ...........................................................................aaaaacccaaGugaacuuuuug............  1      1   seq
    ...........................................................................aaaaacccaaaugaacuuuuugU...........  5      1   seq
    ............................................................................aaaacccGaaugaacuuuuu.............  1      1   seq
    ............................................................................aCaacccaaaugaacuuuuug............  1      1   seq
    ............................................................................aaaacccaaaugaacuuuuug............  12     0   seq
```

Star Mature

```
acuuagcagcagcaguaguccauuguauuugguuggccaaaaaguucauuugggcuuuucuguaauguccuguggaaaaacccaaaugaacuuuuuggccaaccaagca
............................................................................aaaacUcaaaugaacuuuuug............	6	1	seq
............................................................................aaaacccaaaugaacuuuuuU............	1	1	seq
............................................................................aaaacccaaaugaacuuuuuA............	4	1	seq
............................................................................aaaacccaaGugaacuuuuug............	1	1	seq
............................................................................aaaacccGaaugaacuuuuugg...........	2	1	seq
............................................................................aaaacccaaaugaacuuuuugU...........	4	1	seq
............................................................................aaaacccaaaugaacuuuuuggA..........	2	1	seq
.............................................................................aaacccGaaugaacuuuuu.............	28	1	seq
.............................................................................aaacccaaaugaacuuuuug............	2	0	seq
.............................................................................aaacccaaaugaacuuuuugU...........	1	1	seq
.............................................................................aaacccGaaugaacuuuuuggc..........	6	1	seq
.................................................................................ccaaaugaacuuuCuggccaac......	1	1	seq
..................................................................................Uaaaugaacuuuuuggccaac......	1	1	seq
..................................................................................caaaugaacuuuuuggccaacA.....	1	1	seq
```
